# An intronic mutation in *Chd7* creates a cryptic splice site, causing aberrant splicing in a mouse model of CHARGE syndrome

**DOI:** 10.1038/s41598-018-23856-8

**Published:** 2018-04-03

**Authors:** Jacqueline M. Ogier, Benedicta D. Arhatari, Marina R. Carpinelli, Bradley K. McColl, Michael A. Wilson, Rachel A. Burt

**Affiliations:** 1 Murdoch Childrens Research Institute, 50 Flemington Road, Parkville, VIC 3052 Australia; 2 Department of Paediatrics, University of Melbourne, Parkville, VIC 3010 Australia; 3 School of Biosciences, University of Melbourne, Parkville, VIC 3010 Australia; 4 Walter and Eliza Hall Institute, 1G Royal Parade, Parkville, VIC 3052 Australia; 5 ARC Centre of Excellence for Advanced Molecular Imaging, Department of Chemistry and Physics, La Trobe University, Bundoora, VIC 3086 Australia

## Abstract

Alternate splicing is a critical regulator of gene expression in eukaryotes, however genetic mutations can cause erroneous splicing and disease. Most recorded splicing disorders are caused by mutations of splice donor/acceptor sites, however intronic mutations can affect splicing. Clinical exome analyses largely ignore intronic sequence, limiting the detection of mutations to within coding regions. We describe ‘*Trooper*’, a novel mouse model of CHARGE syndrome harbouring a pathogenic point mutation in *Chd7*. The mutation is 18 nucleotides upstream of exon 10 and creates a cryptic acceptor site, causing exon skipping and partial intron retention. This mutation, though detectable in exome sequence, was initially dismissed by computational filtering due to its intronic location. The *Trooper* strain exhibited many of the previously described CHARGE-like anomalies of CHD7 deficient mouse lines; including hearing impairment, vestibular hypoplasia and growth retardation. However, more common features such as facial asymmetry and circling were rarely observed. Recognition of these characteristic features prompted manual reexamination of *Chd7* sequence and subsequent validation of the intronic mutation, highlighting the importance of phenotyping alongside exome analyses. The *Trooper* mouse serves as a valuable model of atypical CHARGE syndrome and reveals a molecular mechanism that may underpin milder clinical presentation of the syndrome.

## Introduction

Alternate splicing is a critical mechanism of gene regulation, with both spliceosomal assembly and catalysis conserved in eukaryotic cells. Simple point mutations causing incorrect mRNA splicing were once considered to be responsible for around 15% of human genetic disease, however computational estimates now suggest that over 50% of genetic disorders are caused by aberrant splicing^1,2^. Additionally, intronic sequence is now understood to contain critical signals such as splice donor (5′), acceptor (3′) and branch sites that define exon/intron boundaries and trigger the correct removal of non-coding sequence from precursor mRNA transcripts to produce mature mRNA. Mutations within consensus sequences can result in cryptic splice site activation, exon skipping and intron retention or frameshift - which in turn affects gene expression and functionality resulting in severe disease, such as CHARGE syndrome.

CHARGE syndrome is a rare human disorder characterized by ocular Coloboma, Heart defects, choanal Atresia, Retarded growth, Genital hypoplasia and Ear anomalies. In most cases, the syndrome is caused by a point mutation within the chromo domain helicase DNA binding protein 7 gene (*CHD7*). Numerous pathogenic mutations in *CHD7* splice sites have been recorded in humans, however, their prevalence is low, which may be due to stringent criteria set in exome analysis pipelines^3–5^. For example, intronic mutations are predominantly considered pathogenic when present within the canonical splice donor–acceptor pair and mutations outside of this intronic region are disregarded as variants of unknown significance. However, a growing body of evidence suggests that even deep intronic mutations are able to severely disrupt gene expression^6^. Herein we describe a murine model of CHARGE syndrome carrying an ethylnitrosourea (ENU) mutagenesis -induced point mutation in *Chd7*. Interestingly, the pathogenic non-canonical splice mutation occurs 16 nucleotides upstream of the 3′ AG splice site, however intron retention and exon skipping occurs. The *Trooper* mouse presents with a mild CHARGE-like phenotype, exhibiting a range of anomalies including growth retardation, hearing-impairment and vestibular hypoplasia. Thus, the *Trooper* mouse is a valuable model of atypical human CHARGE syndrome and the molecular mechanisms that underpin this condition.

## Methods

### Ethics Statement

Procedures were approved by the animal ethics committees of The Walter and Eliza Hall Institute (WEHI) and Murdoch Childrens Research Institute (MCRI) in project numbers 2011.016 and A726 respectively. All experiments conformed to the Australian Code of Practice for the Care and Use of Animals for Scientific Purposes, 8^th^ edition, 2013.

### Mice

Colonies of mice were maintained at the WEHI and the MCRI. Mice housed at MCRI were group-housed in individually ventilated micro-isolator cages (Tecniplast, Buguggiate, VA, Italy) on a 14 hour light/10 hour dark cycle. Mice housed at WEHI were group-housed in individually ventilated micro-isolator cages (Airlaw, Smithfield, NSW, Australia) on a 12 hour light/12 hour dark cycle. Animals were provided with standard Barastoc mouse chow (Ridley AgriProducts, Melbourne, VIC, Australia) and sterilized water *ad libitum*. Environmental enrichment was provided in the form of cardboard toys and sunflower seeds.

### Mutagenesis Screen

Male BALB/c mice were injected intraperitoneally with 85 mg/kg ethylnitrosourea (ENU, Sigma-Aldrich, Castle Hill, NSW, Australia) weekly for 3 weeks as previously described^7^. After a rest period of 12 weeks to recover fertility, treated males were backcrossed with untreated BALB/c females. The resulting progeny (G1) were screened using an acoustic startle response (ASR) test at 8 weeks of age. Mice with an ASR below 200 mV in response to white noise bursts of 115 dB SPL were test-mated to determine heritability of the phenotype. The *Trooper* strain was serially backcrossed to BALB/c for 15 generations prior to phenotypic analysis.

### Genotyping

Genomic DNA was isolated from ear punctures as previously described^8^. Mice were genotyped for the *Chd7 Trooper* mutation using the Amplifluor SNPs HT genotyping system FAM-JOE and Amplifluor Assay Optimization Buffers (S + 25 mM MgCl) (Merck Millipore, Kilsyth, VIC, Australia) using the primers *GAAGGTGACCAAGTTCATGCTGGTCAAATGACTTTAGTTCTGATTT* (forward wild-type), *GAAGGTGACCAAGTTCATGCTGGTCAAATGACTTTAGTTCTGATTA* (forward mutant) and TCATAAGGGAGTGAGCACCA (reverse).

### Acoustic Startle Response

The SR-LAB plug and play ASR system (San Diego Instruments, San Diego, CA, USA) was used to measure the startle response. Testing was conducted in an illuminated environment during the light phase of the lighting cycle. A perspex restraint chamber was used and mice were acclimatised to 70 dB SPL of background white noise for one minute. Clicks were presented in a pre-programmed pseudorandom order and separated by intervals of three to eight seconds. Each mouse underwent six trials of 70, 85, 90, 95 and 100 dB SPL and 16 trials of 115 dB SPL (40 ms white noise click). The largest and smallest recording were deleted before the average startle amplitude was calculated. Prism v 6.0b software was used for data compilation (GraphPad Software Inc, La Jolla, CA, USA).

### Auditory Brainstem Response

An evoked potentials workstation (Tucker Davis Technologies, Alachua, FL, USA) was used to assess the auditory brainstem response (ABR) as previously described^9^. Intraperitoneal injection of 100 mg/kg ketamine and 20 mg/kg xylazine was used and anesthetized mice were kept on a heat pad with eyes protected using REFRESH night time (Allergan, NSW, Australia). A free-field magnetic speaker (model FF1, Tucker Davis Technologies) was placed 10 cm from the left pinna. Computer-generated clicks (100 µs duration, with a spectrum of 0–50 kHz) and 3 ms pure tone stimuli of 4, 8, 16 and 32 kHz were presented with maximum intensities of 100 dB SPL. Subdermal needle electrodes (S06666-0, Rochester Electro-Medical, Inc., Lutz, FL, USA) were positioned at the apex of the skull (+ve), the left cheek (−ve) and the left hind leg (ground). ABRs traces were calculated and analysed using the average of 512 stimuli repeats in BioSig software (Tucker Davis Technologies). The threshold was detected by visual analysis, as the lowest intensity stimulus that reproducibly evoked an ABR.

## Mutation Identification

### Massively Parallel Sequencing

Exome sequencing was completed by the Australian Genome Research Facility (AGRF) using the 100803_MM9_exome_rebal_2_EZ_HX1 exome capture array (Roche Nimblegen, Madison, WI, USA), TruSeq Sample Preparation Kit (Illumina, San Diego, CA, USA) and HiSeq. 2000 Sequencing System (Illumina) using DNA isolated from two N7 *Chd7*^+/*Trooper*^ liver samples. The Bioinformatics Team at the Australian Phenomics Facility then utilised a custom analysis pipeline to align sequence reads to the reference genome (C57BL/6 *NCBI* m37). Raw single nucleotide variant (SNV) calls were filtered and a list of candidate SNVs created as described^10^.

### Data Availability

Deep-sequencing data was deposited into the National Center for Biotechnology Information (NCBI) Sequence Read Archive, SRA accession: SRP126877 (https://www.ncbi.nlm.nih.gov/sra/SRP126877).

### Linkage Mapping

A mapping cohort was produced by intercrossing BALB/c-*Chd7*^+/*Trooper*^ with C57BL/6 mice. At 8 weeks of age, F1 offspring were ABR tested and those with hearing impairment (click threshold >40 dB SPL) were intercrossed, generating 336 F1N1 progeny. Progeny were ABR-tested at 8 weeks of age and euthanized for the collection of tissues. Genomic DNA was isolated from liver sections as previously described^8^. Twenty hearing-impaired F1N1 progeny (click ABR ≥45 dB SPL) were genotyped for 660 SNPs spaced at 5–10 Mb intervals throughout the genome using the iPLEX Gold method^11^, the MassARRAY System Sequenom, San Diego, CA, USA) and an Autoflex MALDI-TOF mass spectrometer (Bruker, Billerica, MA, USA) at AGRF. Haplotypes were aligned in Excel version 14.3.4 (Microsoft, Redmond, WA, USA) for visual identification of shared regions of heterozygosity.

### Sanger sequencing

The *Chd7 Trooper* SNV was PCR-amplified from genomic DNA and sequenced using primers GTCTTGGTCAGGGAAGCAGA and GGATATGAGCTACAGCATTTATTGAA. Capillary separation was performed at the AGRF. Seqman version 10.1 software (DNASTAR, Madison, WI, USA) was used for electropherogram sequence alignment.

### Transcriptome analysis

Mice were euthanized by cervical dislocation. Brain was immediately collected, bisected and placed into RNAlater (Life Technologies) on wet ice. Following a 12 hour incubation at 4°, tissue was disrupted using a mortar and pestle. RNA was extracted using a Quiagen RNEeasy miniKit and cDNA was then synthesized using the SensiFAST™ cDNA Synthesis Kit (Bioline). cDNA was amplified by PCR using primers CTCTCAGAGATTGAGGATGACCT and TTTTTGCACCTGCTCTTCCG and Pfx polymerase (Invitrogen) according to manufacturers recommendations. Purified PCR products were blunt ligated into pBluescript II phagemids digested with EcoRV, and E. coli NEB10-Beta cells transformed with ligation reactions. Colonies containing inserts were identified by blue-white screening, and sequenced using the alternative reverse primer (ACAACCACGTTCAACTCCGT).

### X-ray Micro-Computed Tomography (µCT)

Mice were euthanized by cervical dislocation. Cochleae were dissected from the temporal bones and stored in 4% neutral buffered formalin. μXCT measurements were carried out using an Xradia^©^ micro XCT200 (Carl Zeiss X-ray Microscopy, Inc.), which uses a microfocus X-ray source with a rotating sample holder and an imaging detector system. The source is a closed x-ray tube (tube voltage 40 kV, peak power 10 W). One data acquisition set consisted of 361 equiangular projections over 180 degrees providing a complete tomographic reconstruction. The exposure time was 8 seconds for each projection. The tomographic scan involved rotating the sample whilst recording transmission images on the CCD. Each projection image was corrected for the non-uniform illumination in the imaging system, determined by taking a reference image of the beam without sample. A filtered back-projection algorithm is used to obtain the 3D reconstructed image. The final three-dimensional reconstructed image size was 512 × 512 × 512 voxels with the voxel size of 7.6 µm along each side and Field of View (FOV) of (3.9 mm)^3^. Avizo-6.2 software (Mercury Computer Systems Inc., France) was used for image segmentation. Imaging, 3D modelling and evaluation were performed blinded of the genotype

### Statistical Analysis

Statistical analysis was completed in Prism 6 software (GraphPad Software Inc.). Testing included two-way ANOVAs with post hoc t-tests, Wilcoxon-Mann-Whitney tests and was corrected for repeated testing using the Holm Sidak method.

## Results

The *Trooper* strain emerged in an ENU mutagenesis screen that has produced numerous models of deafness^9,12,13^. G1 founder mice were identified using the ASR test and subsequently bred to validate heritability. The *Trooper* phenotype proved heritable in an autosomal dominant fashion.

### The *Trooper* Phenotype is caused by a mutation in *Chd7*

**Figure 1:**
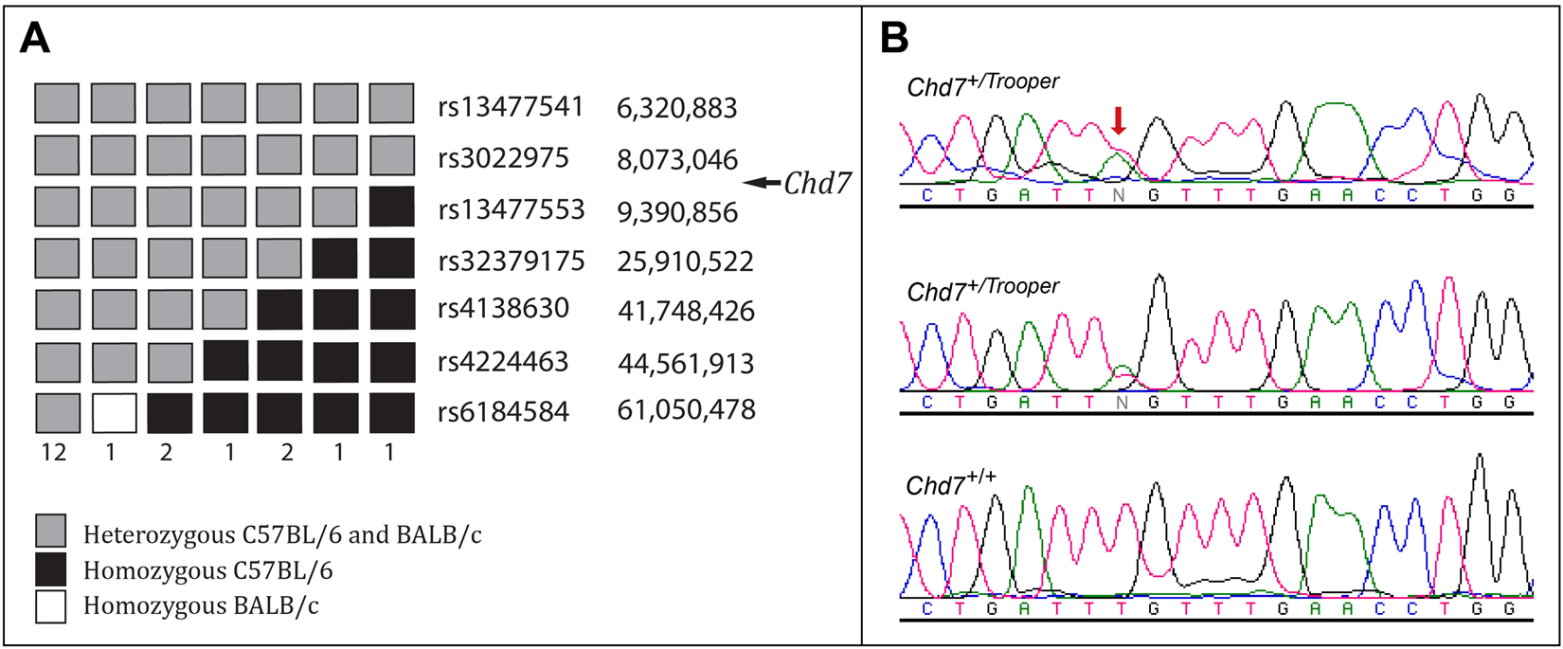
*Trooper* mice harbour an intronic point mutation in *Chd7*. (**A**) 20 F_1_N_1_ mice were classified as hearing impaired following click-ABR testing (threshold ≥45 dB SPL). SNP Haplotypes for the proximal region of chromosome 4 are illustrated, with the numbers below each haplotype representing the number of mice observed with that haplotype. The region between the centromere and rs13477553 was heterozygous in all hearing impaired mice. (**B**) DNA sequence electropherograms of a region of *Chd7* intron 9 in two hearing impaired *Trooper* mice and an unaffected littermate. Both affected mice were heterozygous for a c.3219-18T> A mutation in *Chd7*, which was predicted to affect splicing.

The chromosomal location of the *Chd7* mutation was first identified using meiotic mapping. A cohort of F1N1 mice were grouped as hearing impaired (threshold ≥45 dB SPL) or normal (hearing threshold ≤35 dB SPL) utilising the ABR click test. Twenty hearing impaired F1N1 mice were genotyped for 660 SNPs spaced every 5–10 Mbp throughout the genome. A shared common haplotype was observed proximally on the long arm of chromosome 4, between the centromere and rs13477553 (Fig. 1A). This localized the *Trooper* mutation to a 9Mbp region between the centromere and rs13477553.

Liver derived genomic DNA of two BALB/c-*Chd7*^+/*Trooper*^ N7 mice was subjected to exome enrichment and massively parallel DNA sequencing. Raw single nucleotide variant calls were filtered through a custom analysis pipeline to create a list of candidate SNVs for each mouse. However, no candidate SNVs were returned within the minimal linkage region on chromosome 4. Given that *Chd7* fell within the linkage interval and that *Trooper* mice exhibited similar phenotypic attributes to the previously described *Chd7* ^*Looper*^ mouse^9^, the exome sequence of *Chd7* was re-inspected. Visual examination of sequence alignment data showed that a point mutation within intron 9 of *Chd7* (c.3219-18 T > A, NCBI reference sequence NM_001277149.1) had been detected, but had subsequently been filtered out by the computational pipeline. Sanger sequencing of genomic DNA from hearing impaired and normal littermates validated the SNV (Fig. 1B).

### The Trooper mutation creates a cryptic splice site

**Figure 2:**
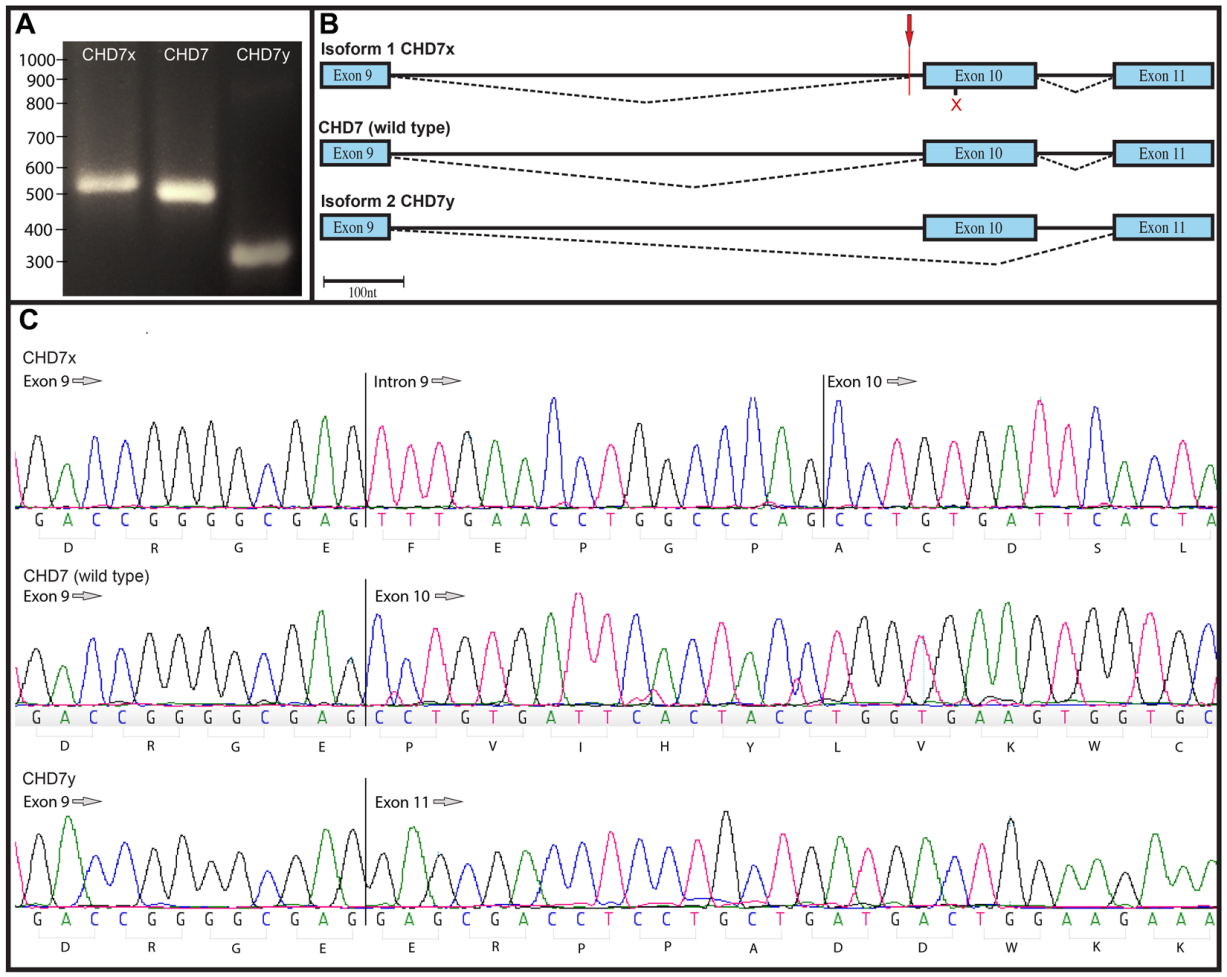
The *Trooper* mutation causes alternate Chd7 transcripts. (**A**) An agarose gel electrophoresis illustrating the three PCR products obtained from the blue/white screen. PCR products were stained using gel red and run through 2.5% agarose for 120 minutes at 80 V. (**B**) A schematic representation of splicing around the *Trooper* mutation in the three amplicons. *Chd7*_x_ retains 16 nt of intron 9 immediately following the mutation site (red arrow), resulting in frame shift which then causes a premature stop codon in exon 10 (red x). The wild type transcript is present as mice are heterozygous for the mutation and the smallest of the transcripts, *Chd7*_y_ is created by the skipping of exon 10 and remains in frame. (**C**) Sequence electropherograms of the three amplicons in the region of the *Chd7 Trooper* mutation.

To investigate transcriptome changes, PCR amplification of *Chd7*^+*/Trooper*^ complimentary DNA was performed. Gel electrophoresis indicated that three transcripts were present. For validation, molecular cloning of the mutant transcripts was performed in a blue/white screen. PCR sequencing of 44 colonies revealed three different transcripts (Fig. 2A), 14 of which had exon 10 skipped, 6 retained part of intron 10 and 24 were wild type (Fig. 2B and C).

### *Trooper* Mice Have Middle Ear defects and Vestibular Organ Hypoplasia

**Figure 3:**
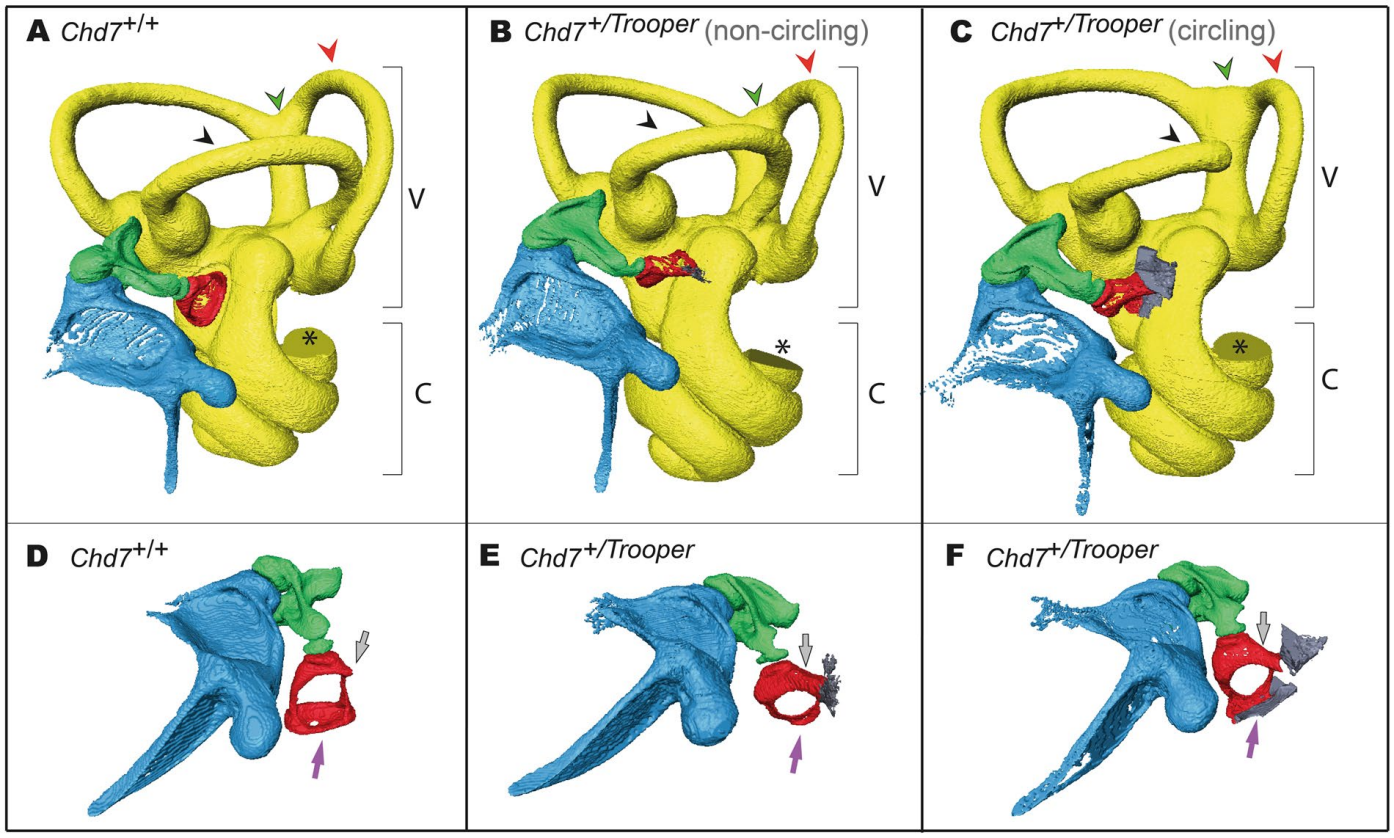
Trooper mice have ossicle and semicircular canal malformations. (**A–C**) X-ray μCT images of the left middle and inner ears of *Chd7*^+*/*+^and *Chd7*^+*/Trooper*^ mice including the vestibular apparatus (V) and cochlea (C). The anterior canal (red arrowhead) is hypoplastic and the intersection of the posterior and superior canals (green arrowhead) is enlarged and misshapen in both *Chd7*^+*/Trooper*^ mice when compared with a *Chd7*^+*/*+^control. (**B**) In the non-circling *Chd7*^+*/Trooper*^ mouse the lateral semicircular canal (black arrowhead) is complete but hypoplastic. (**C**) In the circling *Chd7*^+*/Trooper*^ mouse the lateral semicircular canal (black arrowhead) is incomplete. The missing section in each cochlea (*) is an artifact of the imaging/reconstruction process. n = 1 *Chd7*^+*/*+^ and 2 *Chd7*^+*/Trooper*^ mice. (**D,E**) An alternate view of the above middle ears. The *Chd7*^+*/Trooper*^ stapes was rounded and small with an unusual bone growth extending from the tubercle to the otic capsule (grey arrow) and the stapes footplate (purple arrow) was also deformed. In the non-circling *Trooper*, the footplate was under developed and not contacting the oval window however the more severely affected *Chd7*^+*/Trooper*^ (with circling behavior) has stapedial fusion with the oval window.

To assess *Trooper* mice for phenotypes associated with CHARGE syndrome, the middle and inner ears were imaged using µCT. This was done on *Trooper* mice with and without circling behavior. The *Chd7*^+*/Trooper*^ stapes was malformed in a small rounded shape and an unusual bone growth extended between the tubercle and otic capsule. In the non-circling mouse, µCT imaging showed that the stapes footplate was deformed and did not contact the oval window (Fig. 3B). However, a more severely affected *Chd7*^+*/Trooper*^ (with circling behavior) had the stapedial footplate fused to the oval window (Fig. 3C). µCT also showed partial development of the lateral semicircular canal and varied levels of posterior and anterior canal hypoplasia. The intersection of the posterior and superior canals is enlarged and misshapen.

In CHARGE affected individuals, hypoplasia of the semicircular canals can cause vestibular areflexia, resulting in poor motor and speech development^14^. In mice, this impairment manifests in head bobbing, tilting and circling behaviors^15^. These traits were observed in *Trooper* mice indicating that their vestibulo-ocular reflex is impaired. However, a full vestibular evaluation was not performed.

### *Trooper* mice are hearing impaired

**Figure 4:**
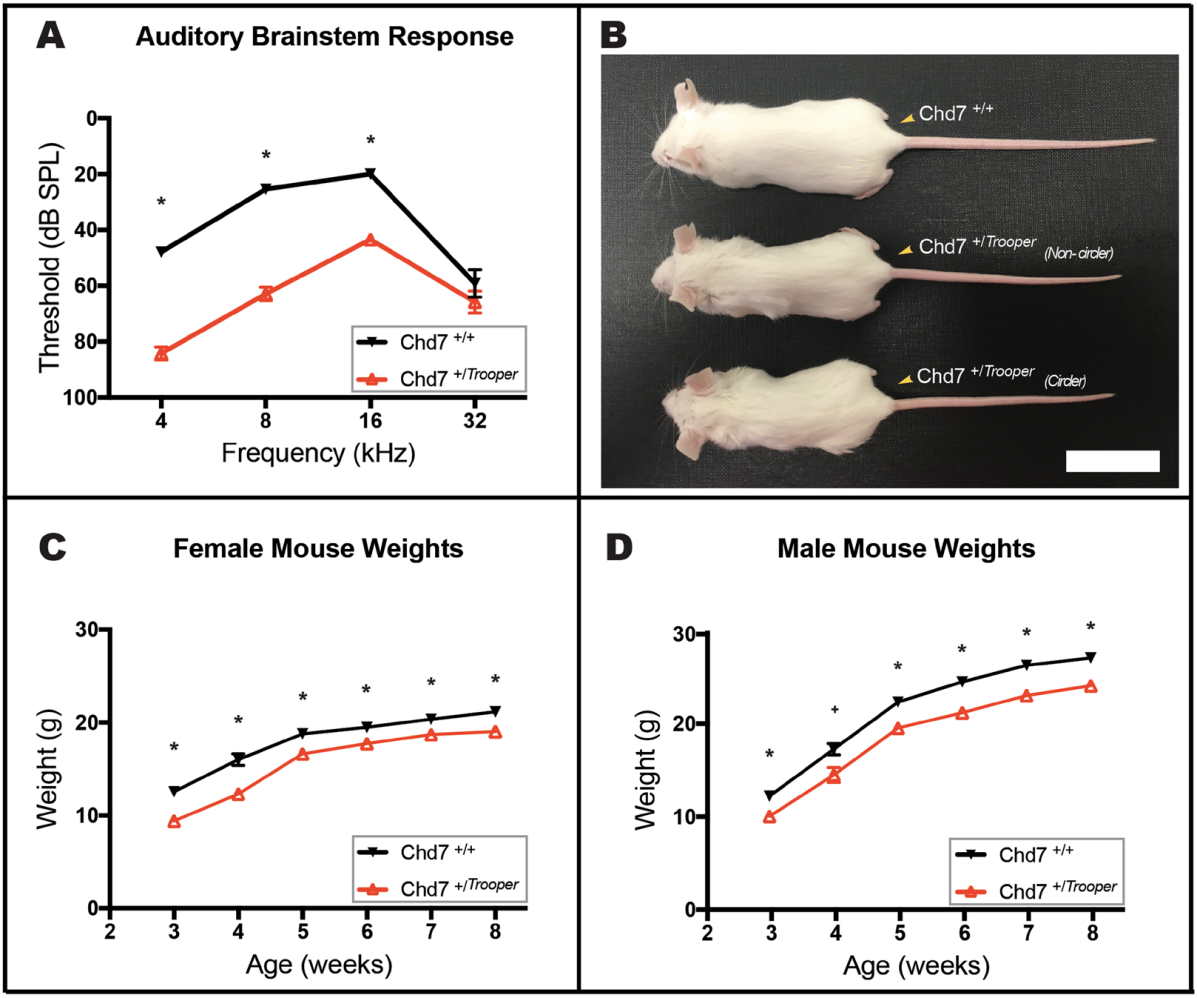
Trooper mice are hearing impaired and have growth deficiencies. (**A**) Average ABR thresholds of *Chd7*^+*/Trooper*^ (n = 12) and *Chd7*^+*/*+^ (n = 12) mice. *Chd7*^+*/Trooper*^ thresholds were significantly elevated at 4,8 and 16 kHz in comparison to *Chd7*^+*/*+^ controls. *p < 0.001 based on t- tests performed with the Holm-Sidak correction for multiple comparisons. Error Bars = SEM. (**B**) Photograph of female *Chd7*^+/*Trooper*^ and *Chd7*^+*/*+^ littermates (at 50 days of age) illustrating the reduced size and length of *Chd7*^+/*Trooper*^ mice. Scale Bar = 3 cm. (**C,D**) Average weights of male and female *Chd7*^+*/*+^ (n = 19 female and 20 male) and *Chd7*^+*/Trooper*^ (n = 26 female and 21 male) mice, from 3–8 weeks of age.

ABR thresholds of *Chd7* ^+*/Trooper*^ mice were significantly elevated between 4 and 16 kHz when compared with wild type controls (Fig. 4A). Hearing impairment was greatest at lower frequencies, with an average threshold shift of 35–40 dB SPL at 4 and 8 kHz. An average threshold shift of 20 dB was observed at 16 kHz. At 32 kHz, *Chd7*^+*/Trooper*^ mice tended to have slight hearing threshold elevation, however this was not significant.

### The *Trooper* mutation is homozygous lethal

*Chd7*^+*/Trooper*^ mice crossed with BALB/c mice produced 277 progeny consisting of 148 *Chd7*^+/+^ (66 f, 82 m) and 129 *Chd7*^+*/Trooper*^ (71 f, 58 m). We expected a 1:1 sex and genotype ratio and no significant deviation from this distribution was observed (two tailed P = 0.224, using the Chi Square test). Intercrossing of *Chd7*^+*/Trooper*^ mice produced 39 offspring, none of which were homozygous for the mutation (deviating significantly from the expected distribution-two tailed P = 0.002, using the Chi Square test). 18 mice were *Chd7*^+*/*+^ (9 f, 9 m) and 21 were *Chd7*^+*/Trooper*^ (14 f, 7 m), indicating that homozygotes were dying embryonically. Heterozygous mice were fertile and reasonably healthy, but displayed growth deficiency (Fig. 4B–D).

## Discussion and Conclusions

**Figure 5:**
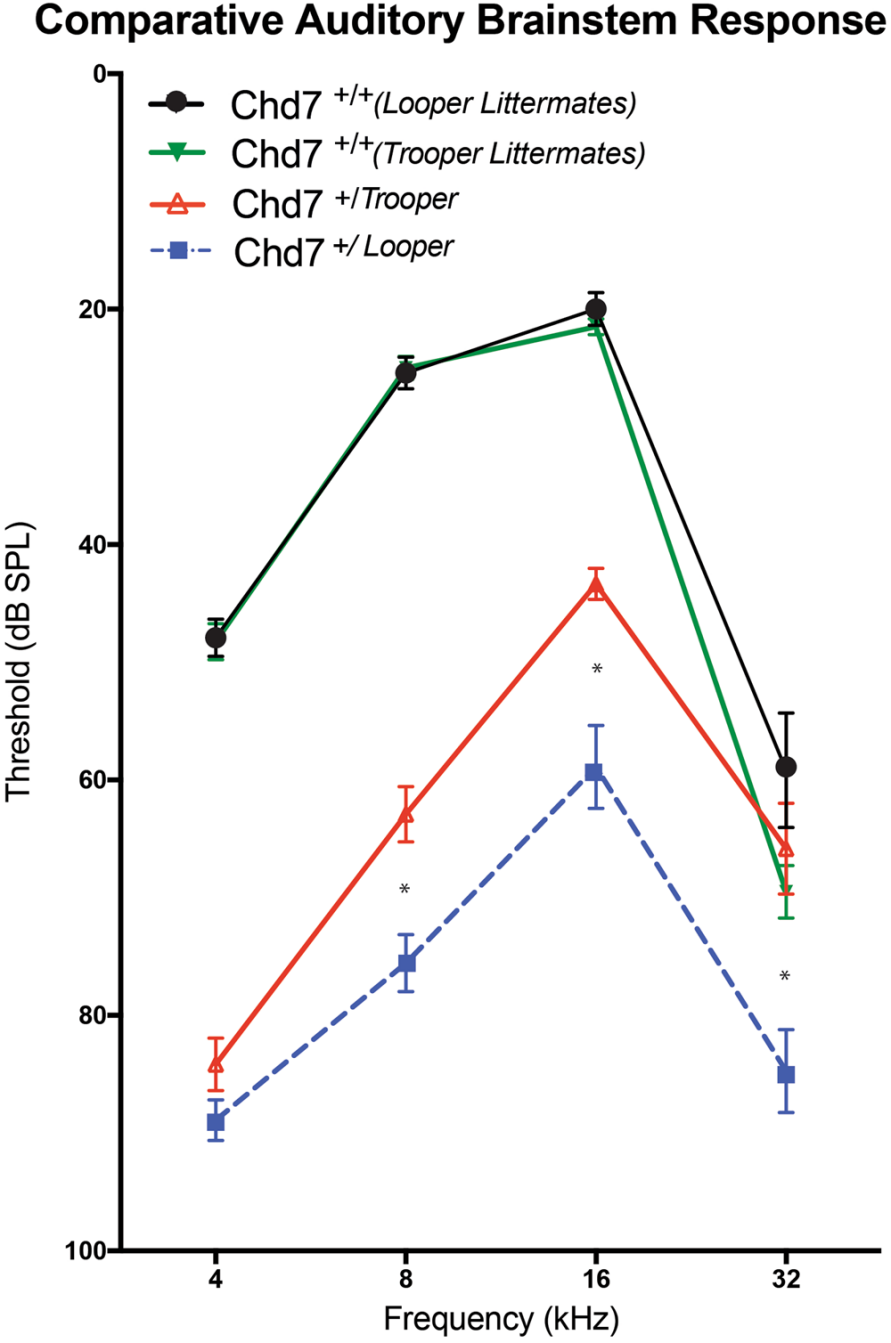
*Trooper* mice have a milder hearing phenotype than the previously reported *Looper* strain. Average ABR thresholds of *Chd7*^+*/Trooper*^, *Chd7*^+*/Looper*^, and their *Chd7*^+*/*+^ controls. The hearing of *Trooper* mice is on average, significantly better than *Looper* mice (*p < 0.002 based on t- tests performed with the Holm-Sidak correction for multiple comparisons). *Chd7*^+/*Looper*^ and the corresponding *Chd7*^+*/*+^ control cohort were previously published in *Ogier et al*.^9^).

The *Trooper* mutation emerged from an ENU mutagenesis screen designed to identify hearing impaired mice. Linkage mapping, exome sequencing and Sanger sequencing were used to pinpoint a mutation (c.3219-18T > A) in the *Chromodomain Helicase DNA binding 7* (*Chd7*) gene. Mutations in this gene cause a pattern of defects collectively known as CHARGE syndrome in humans. CHARGE is a complex disease, affecting multiple organs with extreme heterogeneity between individuals. Likewise, variable phenotypic penetrance is common amongst the numerous murine CHARGE models. CHD7 deficient mouse lines are typically named after their striking circling behavior. Examples such as *Cyclone*, *Dizzy*^16^, *Whirligig*^17^and *Looper*^9^ have marked vestibular deformity, severe eye anomalies, choanal defects, cleft palate and multiple minor CHARGE features. On the other hand, tail chasing is essentially absent from the *Trooper* strain. Choanal atresia and heart defects are unlikely given the survival rates of *Chd7*^+*/Trooper*^ mice post weaning, and common pathologies such as micropthalmia and blepharoconjunctivitis were rarely observed (Table 1). Additionally *Chd7*^+*/Trooper*^ hearing loss was less severe than in other strains (Fig. 5)^9^. Overall, the *Trooper* phenotype is particularly mild (Table 1). Like *Trooper*, the atypical CHARGE phenotype in humans lacks major diagnostic features such as heart defects and choanal atresia. However hearing loss, growth deficiency and inner ear malformations remain common^18^. The consistency of inner ear malformation across all forms of CHARGE syndrome highlights that structural patterning in the ear is particularly sensitive to CHD7 expression levels. Indeed, CHD7 knockdown inhibits neural crest cell migration into the pharyngeal arches, impacting derived structures such as the ossicle chain^19^. To date, functional analysis of *CHD7* has proven difficult, due to the exceptional size of the gene (185Kbp, with 42 exons, GRCh38.p7, NC_000008.11, protein ~ 336 kDa). As a result, there is no clear explanation as to why some patients present with a milder form of the disease, however it is thought that *CHD7* truncating mutations cause a more severe phenotype than missense mutations^20^. CHD7 deficiency does affect cellular differentiation, proliferation and migration in a dose specific manner, which may account for some of the milder CHARGE phenotypes^19^. Yet, a genotype-phenotype correlation does not exist. This is most clearly evident when multiple sibling pairs (including monozygotic twins) carrying identical mutations present with significant phenotypic differences^5^.

**Table 1:** Penetrance of *Trooper* pathology compared with *Looper* pathology.

Phenotype	*Chd7 Trooper*	*Trooper* WT	*Chd7 Looper*	*Looper* WT
Circling	4%	0%	83%	0%
	(2/52)	(0/46)	(5/6)	(0/10)
Hearing impairment	100%	0%	100%	0%
	(12/12)	(0/12)	(18/18)	(0/22)
Semicircular Canal malformation	100%	0%	100%	0%
	(2/2)	(0/1)	(3/3)	(0/1)
Blepharoconjunctivitis	12%	0%	69%	0%
	(6/52)	(0/46)	(11/16)	(0/19)
Post Weaning Survival (of total colony population)	53%	47%	41%	59%
	(52/98)	(46/98)	(68/167)	(99/167)

The *Trooper* phenotype is milder than previously reported *Chd7* mouse mutants.

The *Trooper* mutation creates an AG marker within a cryptic splice site, that interrupts canonical splicing of *Chd7*. In eukaryotes, dinucleotides at the 5′ (GT) and 3′(AG) ends of intronic sequence are essential for specifying splice site positions. These markers define exon boundaries and lead to the recruitment of the spliceosome for intron removal. Multiple cryptic splice sites exist within precursor-mRNA’s, however they are not used unless the authentic splice site is interrupted by mutation. In rarer cases, such as the *Trooper* mutation, intronic de novo 3′ splice sites are activated by the creation of an AG marker in the polypyrimidine tract^21^. Generally, activated cryptic splice sites are within 100 nucleotides and upstream of the authentic splice site^22^. The *Trooper* mutation is consistent with these observations and is preceded by a pyrimidine rich sequence, which likely initiates spliceosome assembly. Thus, the *Chd7*^+/*Trooper*^ mutation activates a cryptic splice site, prematurely signaling the end of intron 9. As a result, two aberrant isoforms are produced.

The isoform we termed *Chd7*_x_ retains 16 nucleotides from the 3′ end of intron 9. This insertion causes a frame shift, leading to a premature stop codon in Exon 10. CHD7_x_ terminates before the helicase, BRK and SANT domains and is presumed to be non functional. The predicted truncation may induce nonsense mediated mRNA decay. However, the presence of CHD7_x_ cDNA in the blue/white screen shows that the product was not completely degraded prior to reverse transcription.

The second transcript, *Chd7*_y_, skips Exon 10. Skipping of Exon 10 removes a large portion of the second of two chromo domains in CHD7 required for histone tail binding. However, remaining in frame, *Chd7*_*y*_ likely avoids mRNA degradation. It is certainly possible that *Chd7*_*y*_ retains some function and given the *Trooper* phenotype, a dominant negative effect is unlikely.

The atypical CHARGE pathology observed in *Chd7*^+*/Trooper*^ mice indicates that one of the alternate isoforms is retaining a measure of functionality, or that the spliceosome is occasionally creating enough wild type CHD7 to reach a critical threshold. As the wild type *Chd7* transcript was not significantly over represented in the blue/white screen, we predict that CHD7_*y*_ is retaining partial functionality with a single chromodomain.

Chromodomains are highly conserved, structural components of large chromatin remodeling proteins that regulate gene activity and genome organization. In a protein specific manner, chromodomains present in tandem, individually or with related ‘chromo shadow domains’ ^23^ A defining characteristic of Chromodomain Helicase DNA binding proteins is the presence of dual chromodomains. Whilst the mechanism of H3 histone binding in chromatin remodelers differs significantly between proteins containing single and dual chromodomains, the efficacy of H3 binding is comparable^24^. Therefore CHD7_*y*_ will likely retain histone binding capabilities despite deletion of the C-terminal chromodomain. A human CHD7 splice variant (CHD7s) provides further evidence that CHD7_*y*_ may be functional without tandem chromodomains. CHD7s lacks the C-terminal chromodomain as well as the helicase/ATPase, DNA-binding, and BRK domains. CHD7s acts cooperatively with CHD7 in the nucleus and antagonizes CHD7 in the nucleoplasm^25^.The ability of CHD7s to drive 45S rRNA gene transcription indicates that the CHD7 N-terminal chromodomain is functional in the absence of the CHD7 C-terminal chromodomain. Further analysis of the *Trooper* CHD7 isoforms will likely establish the functional importance of chromodomains within a tandem repeat.

There is mounting evidence of the significance of RNA splicing with regard to human disease, which suggests the practice of filtering out intronic variants (of unknown significance) is limiting our ability to diagnose patients. Currently, as many as 30% of CHARGE patients lack a genetic diagnosis. Some chromosomal deletions, one *SEMA3E* mutation, one *RERE* duplication and one *KMT2D* mutation have reportedly caused CHARGE like attributes^26–28^. However *CHD7* mutations are the preponderant cause of clinically diagnosed CHARGE^27^. We postulate that the poor genetic diagnosis rate is due to methodology in diagnostic laboratories, which tend to limit *CHD7* analysis to coding exons and dinucleotide splice sites^3,5^. Certainly, the *Trooper* mutation would be missed with current diagnostic methods and as the 48 nt leading to exon 10 in *Chd7* is conserved between species, the mutation could be pathogenic in humans (NCBI ref seq. NG_007009.1). The sequence conservation of this region also indicates functional relevance beyond the canonical splice site.

After projects derived from the Encyclopedia of DNA Elements (ENCODE) contentiously suggested that 80% of the human genome had biochemical functions, a wealth of knowledge has been gained showing that the evolutionarily dynamic intronic sequence is biologically relevant in humans^29–31^. In an age of rapidly evolving diagnosis via genetic testing, the *Trooper* mouse is a timely reminder that as the methodology around exome and genome sequence analysis develops, it would be wise to reconsider ways to screen for mutations in introns, particularly in sequence with homology to cryptic splice sites.

## Conclusion

*Trooper* mice carry an intronic ENU –induced point mutation that results in alternate splicing of *Chd7*. The strain has a mild phenotypic presentation resembling atypical CHARGE syndrome including hearing impairment, hypoplasia of the semicircular canals and stapes malformation. One of the *Chd7* alternate transcripts detected in *Trooper* is predicted to be non-functional whilst the other is likely translated, with the resultant protein at least partially functional. The combined expression of CHD7 and CHD7_y_ in *Trooper* relative to the haploinsufficiency observed in other models such as *Looper* likely explains the milder presentation of the phenotype.

Further study of the *Trooper Chd7* isoforms will provide insight into the functional domains of CHD7 and clarify the dosage effects of CHD7 expression on development and function in a variety of organ systems. In addition, the identification of this mutable cryptic splice site within the intronic sequence of *Chd7* should prompt closer scrutiny of the non-coding genomic sequence of CHARGE patients for whom coding mutations of CHD7 have not been identified.

## Electronic supplementary material

Supplementary Figure 1

## References

[1] Krawczak M, Reiss J, Cooper DN (1992). The mutational spectrum of single base-pair substitutions in mRNA splice junctions of human genes: causes and consequences. Human Genetics.

[2] Lopez-Bigaz N, Audit B, Ouzounis C, Parra G, Guigo R (2005). Are splicing mutations the most frequent cause of hereditary disease?. FEBS Lett.

[3] Bartels CF, Scacheri C, White L, Scacheri PC, Bale S (2010). Mutations in the CHD7 gene: the experience of a commercial laboratory. Genet Test Mol Biomarkers.

[4] Kim H-G (2008). Mutations in CHD7, encoding a chromatin-remodeling protein, cause idiopathic hypogonadotropic hypogonadism and Kallmann syndrome. Am J Hum Genet.

[5] Jongmans MCJ (2006). CHARGE syndrome: the phenotypic spectrum of mutations in the CHD7 gene. Journal of Medical Genetics.

[6] Vaz-Drago R, Custodio N, Carmo-Fonseca M (2017). Deep intronic mutations and human disease. Hum Genet.

[7] Bode VC (1984). Ethylnitrosourea mutagenesis and the isolation of mutant alleles for specific genes located in the T region of mouse chromosome 17. Genetics.

[8] Laird PW (1991). Simplified mammalian DNA isolation procedure. Nucleic Acids Res.

[9] Ogier JM (2014). CHD7 deficiency in ‘Looper’, a new mouse model of CHARGE syndrome, results in ossicle malformation, otosclerosis and hearing impairment. PLoS One.

[10] Andrews TD (2012). Massively parallel sequencing of the mouse exome to accurately identify rare, induced mutations: an immediate source for thousands of new mouse models. Open Biol.

[11] Mendisco F (2011). Application of the iPLEX Gold SNP genotyping method for the analysis of Amerindian ancient DNA samples: benefits for ancient population studies. Electrophoresis.

[12] Carpinelli MR, Manning MG, Kile BT, Burt RA (2013). Two ENU-induced alleles of Atp2b2 cause deafness in mice. PLoS One.

[13] Carpinelli MR (2014). A new mouse model of Canavan leukodystrophy displays hearing impairment due to central nervous system dysmyelination. Dis Model Mech.

[14] Admiraal RJ, Huygen PL (1997). Vestibular areflexia as a cause of delayed motor skill development in children with the CHARGE association. Int J Pediatr Otorhinolaryngol.

[15] Vidal P-P, Degallaix L, Josset P, Gasc J-P, Cullen KE (2004). Postural and locomotor control in normal and vestibularly deficient mice. J Physiol.

[16] Nolan, P. M. *et al*. A systematic, genome-wide, phenotype-driven mutagenesis programme for gene function studies in the mouse. *Nat Genet* **25**, 440–443 (2000). 10.1038/78140 10932191

[17] Hawker, K., Fuchs, H., Angelis, M. H. & Steel, K. P. Two new mouse mutants with vestibular defects that map to the highly mutable locus on chromosome 4. **Int J Audiol****44**, 171–177 (2005). 10.1080/14992020500057434 15916118

[18] Hale CL, Niederriter AN, Green GE, Martin DM (2016). Atypical phenotypes associated with pathogenic CHD7 variants and a proposal for broadening CHARGE syndrome clinical diagnostic criteria. American journal of medical genetics. Part A.

[19] Bajpai R (2010). CHD7 cooperates with PBAF to control multipotent neural crest formation. Nature.

[20] Marcos S (2014). The prevalence of CHD7 missense versus truncating mutations is higher in patients with Kallmann syndrome than in typical CHARGE patients. J Clin Endocrinol Metab.

[21] Kralovicova J, Christensen MB, Vorechovsky I (2005). Biased exon/intron distribution of cryptic and de novo 3′ splice sites. Nucleic Acids Res.

[22] Nakai K, Sakamoto H (1994). Construction of a novel database containing aberrant splicing mutations of mammalian genes. Gene.

[23] Aasland R, Stewart AF (1995). The chromo shadow domain, a second chromo domain in heterochromatin-binding protein 1, HP1. Nucleic Acids Res.

[24] Flanagan JF (2005). Double chromodomains cooperate to recognize the methylated histone H3 tail. Nature.

[25] Kita Y, Nishiyama M, Nakayama KI (2012). Identification of CHD7S as a novel splicing variant of CHD7 with functions similar and antagonistic to those of the full-length CHD7L. Genes Cells.

[26] Jordan, V. K. *et al*. Genotype-phenotype correlations in individuals with pathogenic RERE variants. *Hum Mutat* Epub ahead of print, 10.1002/humu.23400 (2018). 10.1002/humu.23400 PMC5903952 29330883

[27] Sanlaville D, Verloes A (2007). CHARGE syndrome: an update. Eur J Hum Genet.

[28] Badalato L (2017). KMT2D p.Gln3575His segregating in a family with autosomal dominant choanal atresia strengthens the Kabuki/CHARGE connection. Am J Med Genet A.

[29] ENCODE Project Consortium (2012). An integrated encyclopedia of DNA elements in the human genome. Nature.

[30] Patrushev LI, Kovalenko TF (2014). Functions of noncoding sequences in mammalian genomes. Biochemistry (Mosc).

[31] Rands CM, Meader S, Ponting CP, Lunter G (2014). 8.2% of the Human genome is constrained: variation in rates of turnover across functional element classes in the human lineage. PLoS Genet.

