# Supplementary material for: An intronic mutation in Chd7 creates a cryptic splice site, causing aberrant splicing in a mouse model of CHARGE syndrome
Source: Sci Rep. 2018 Apr 3;8:5482. doi: 10.1038/s41598-018-23856-8 (PMC5882948; doi:10.1038/s41598-018-23856-8)
Supplement: Supplementary file 1 — Supplementary Figure 1 [file 41598_2018_23856_MOESM1_ESM.pdf]

An intronic mutation in *Chd7* creates a cryptic splice site, causing aberrant splicing in a mouse model of CHARGE syndrome.

Jacqueline M Ogier, Benedicta D Arhatari, Marina R Carpinelli, Bradley K McColl, Michael A Wilson, Rachel A Burt.

<u>Supplementary information</u>

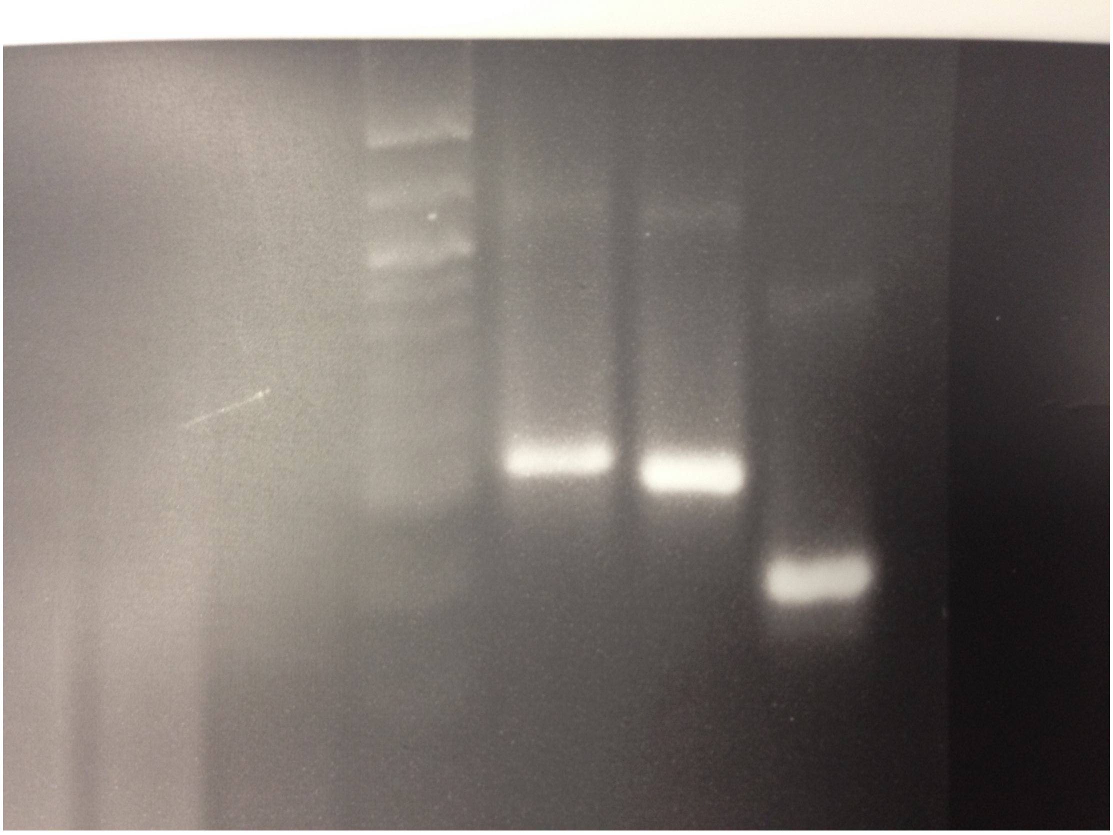

Supplementary Figure 1: Un-cropped version of the image presented in Figure 2A of the main text (An agarose gel electrophoresis illustrating the three PCR products obtained from the blue/white screen.) Gel imaged using a Syngene G:BOX gel doc in conjunction with Syngene GeneSys software. Image initially printed using a Mitsubishi P95 monochrome printer and final image as shown taken using an Apple iPhone 4.
